# The Role of Haptoglobin Polymorphism in Cardiovascular Disease in the Setting of Diabetes

**DOI:** 10.3390/ijms22010287

**Published:** 2020-12-30

**Authors:** Shmuel Somer, Andrew P. Levy

**Affiliations:** Rappaport Faculty of Medicine, Technion-Israel Institute of Technology, 1 Efron st. Bat Galim, Haifa 3525433, Israel; ssomer@gmail.com

**Keywords:** cardiovascular disease, diabetes mellitus, haptoglobin, hemoglobin, vitamin E, glycemic control

## Abstract

Atherosclerotic cardiovascular disease (CVD) is the major cause of morbidity and mortality in individuals with diabetes mellitus (DM). Preclinical models have suggested that excessive oxidative stress and hyperglycemia are directly responsible for this pathological association. However, numerous clinical trials involving the administration of high doses of the antioxidant vitamin E or attempts at strict glycemic control have failed to show a significant reduction of CVD in DM patients. We describe here a possible explanation for the failure of these trials, that being their lack of proper patient selection. The haptoglobin (Hp) genotype is a major determinant of the risk of CVD in the setting of DM. Treatment of individuals with the high-risk Hp genotype with antioxidants or aggressive glycemic control has shown benefit in several small studies. These studies suggest a precision medicine-based approach to preventing diabetes complications. This approach would have a profound effect on the costs of diabetes care and could dramatically reduce morbidity from diabetes.

## 1. Introduction

Diabetes Mellitus (DM) patients are at increased risk for the development of cardiovascular disease (CVD) [1]. Increased oxidative stress and the hyperglycemia associated with DM have been proposed to be contributing factors to this increased risk of CVD in individuals with DM [2]. Accordingly, strategies designed to reduce oxidative stress and hyperglycemia have been investigated in numerous randomized clinical trials aimed at reducing CVD in DM [3,4,5]. However, virtually all of these studies using the antioxidant vitamin E or strict glycemic control have not only shown a lack of improvement but even suggested harm to those DM patients who received these interventions [6]. One possible explanation for the failure of these studies may be the lack of proper patient selection in determining who would receive these interventions [3,7]. We are proposing that with the correct selection of patients with the haptoglobin (Hp)2-2 genotype, these therapies could potentially reduce the risk of CVD in the setting of DM.

There are two common Hp alleles, denoted 1 and 2, at the Hp genetic locus [8,9]. Haptoglobin is a major plasma protein that functions by binding and clearing free hemoglobin (Hb) to reduce its toxicity [10]. Accordingly, there are three possible Hp genotypes: Hp1-1, Hp2-1, and Hp2-2 [6]. The estimated frequencies of the genotypes are 15–18% for Hp1-1, 46% for Hp2-1, and 38% for Hp2-2 [11]. In Israel, 49% of the population has an Hp2-2 genotype. In India, 90% of the population presents the Hp2-2 genotype [12]. In this literature review, we aim to discuss all published interventional studies assessing the ability of vitamin E or aggressive glycemic control to reduce the risk of CVD in patients with the Hp2-2 genotype. 

## 2. Studies Assessing CVD in Relation to the Haptoglobin Genotype 

More than 15 longitudinal studies have assessed the relationship between the Hp genotype and incident CVD in the setting of DM. Collectively, in over 10,000 DM individuals, these studies have demonstrated after further analysis that the Hp2-2 genotype is strongly predictive of increased CVD in DM, as seen in Table 1 [13]. The Heart Outcome Prevention Evaluation (HOPE) study assessed the effects of angiotensin-converting enzyme (ACE) inhibition and vitamin E on incident cardiovascular disease (CV death, stroke, and non-fatal myocardial infarction (MI)) [14]. This trial tested 9541 patients, all aged 55 years or older, who were considered to be at high risk for CVD, including DM patients. The patients were randomized according to a two-by-two factorial design to receive either vitamin E treatment or a matching placebo and either an ACE inhibitor (Ramipril) or its matching placebo. In the vitamin E arm of the study, the incidence of the primary study outcome of the combination of CV death, MI, and stroke was not significantly different between patients who received vitamin E and those who received the placebo. In the Ramipril arm of the study, on the other hand, a significant benefit was shown for Ramipril on CV endpoints [15]. The Strong Heart Study (SHS) focused on a group of American Indians. It was designed to estimate CVD mortality and morbidity rates and the prevalence of CVD risk factors in this specific population. The study population consisted of 12 tribes in areas near Arizona and southern Oklahoma and in Aberdeen surroundings of North and South Dakota [16]. The Munich Post-PCA study consisted of several thousand diabetic and nondiabetic patients who had previous coronary syndromes and who underwent percutaneous transcatheter coronary angioplasty (PTCA). Serum and DNA were collected from this cohort on presentation, with the intent of using this material to identify possible markers that would predict the need for an additional revascularization procedure and/or to repeat PTCA of the original responsible lesion [17]. The Israel Cardiovascular Vitamin E (ICARE) study, involving 47 primary health care clinics in the Haifa area, assessed the association of the Hp genotype with incident CVD in over 3000 individuals. Additionally, in ICARE the 1434 Hp2-2 individuals were randomized to vitamin E or placebo in an attempt to reduce the expected increased incidence of CVD in the Hp 2-2 group [18]. The Epidemiology of Diabetes Complications (EDC) study consisted of Type I diabetes patients. This study sought to understand the association between serum (such as hemoglobin (Hb)A1c) and genetic markers predisposing to the development of CVD in Type I diabetes patients [19]. The Women’s Health Study (WHS) focused on the prevention of CVD and cancer. They tested women aged 45 years and older with no previous history of coronary heart disease (CHD) and satisfying other criteria, treating one group with aspirin vs. placebo and another group with vitamin E vs. placebo. The data from the trial showed no overall benefit for major cardiovascular events or other related diseases [20]. The Diabetes Control and Complications Trial/Epidemiology of Diabetes Interventions and Complications (DCCT/EDIC) study tested 1441 subjects with type 1 DM, comparing the effect of intensive therapy with that of conventional therapy on the incidence of microvascular and macrovascular complications. Intensive therapy significantly lowered HbA1c levels to approximately 7%, while the conventionally treated group presented HbA1c levels of 9%. During the study, greater glucose lowering was associated with a significant decrease in microvascular complications of diabetes, but the incidence of CVD events was too small to make any conclusions. However, 10 and 25 years after the trial had ended, those individuals in the DCCT who had received more intensive therapy had significantly fewer CVD events compared to the group which had been randomized to conventional therapy, during the active phase of the study [21]. The Nurses’ Health Study (NHS) involved a cohort from 1980 consisting of female nurses who participated as the baseline group of the study. Since the first study, many investigations have been carried out on this group, including on the risk of cardiovascular disease [22]. The Health Professionals Follow-Up Study (HPFS) began in 1986 to evaluate a series of hypotheses about men’s health related to nutrition. It was designed as a follow-up to the Nurses’ Health Study. It included over 50,000 male health professionals between the ages of 40 and 75. As in the Nurses’ Health Study, the participants of the HPFS completed questionnaires every two years regarding their disease diagnoses, disease risk factors, drug use, and lifestyle characteristics [23]. The Bruneck Study, which was launched in the summer of 1990, included a random sample of 1000 men and women. The investigators evaluated disease epidemiology and many other factors with a focus on atherosclerosis, CVD, aging and longevity, neurological diseases, disorders of the bone, and cancer [24]. In all these trials, Hp typing was done on stored serum or plasma specimens collected at the onset of the trial from the participants. 

## 3. Mechanism by Which the Hp Genotype May Impact the Risk of CVD

**Figure 1:**
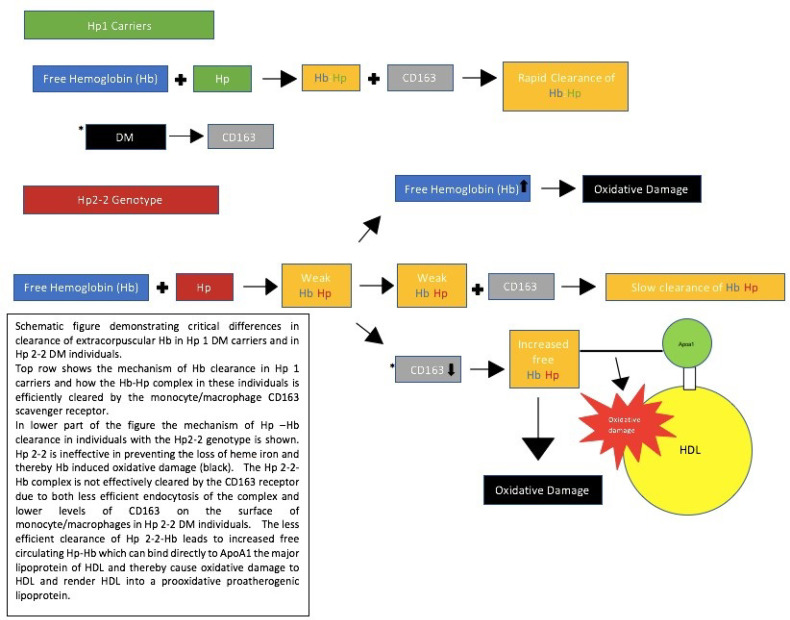
Demonstration of differences in the clearance of extracorpuscular hemoglobin. Hp, haptoglobin, Hb, hemoglobin, DM, diabetes mellitus, HDL, high-density lipoproteins [27].

The studies presented in Table 1 demonstrate that DM patients with the Hp2-2 genotype have an up-to-five-time increased risk of CVD compared to DM individuals with the Hp1-1 genotype, while Hp2-1 individuals have an intermediate risk [14]. The mechanism whereby the Hp genotype confers a risk for CVD is a direct consequence of the differences in function between Hp proteins resulting from the different Hp genotypes. The function of Hp is to bind to Hb that is released during intravascular destruction of erythrocytes (approximately 6 g of Hb per day). This extracorpuscular “free” Hb is a potential important oxidant, and the binding of Hp to free Hb prevents, in large part, the oxidative action of Hb [25]. The monocyte/macrophage receptor CD163 binds the Hp–Hb complex, thereby clearing extracorpuscular, free Hb from the blood [26]. However, the Hp 2-2 protein is deficient in its ability to prevent Hb-induced oxidation and in its ability to promote the clearance of Hb–Hp by the CD163 receptor. These deficiencies result in greater oxidative stress in Hp 2-2 individuals, which is further enhanced in the setting of DM due to the ability of DM to potentiate the oxidative potential of Hb and to downregulate CD163 expression, as shown in Figure 1 [27,28,29]. As a result of the impaired clearance of Hp–Hb 2-2 complexes in DM patients, there is an increased concentration of Hp–Hb in Hp 2-2 DM individuals, which allows the complex to associate with other plasma proteins to which it normally does not bind (such as low-density lipoproteins (HDL)) [30]. Hp can bind directly to ApoA1, the major apolipoprotein of HDL, and thereby tether Hb to which it is complexed to HDL. This tethering transforms the normally antiatherogenic and antioxidative HDL particle into a proatherogenic prooxidative dysfunctional HDL particle, due to its Hb cargo, [3,31].

## 4. Vitamin E Treatment Provides Protection against CVD in DM Patients with the Hp2-2 Genotype

Steinberg and colleagues originally proposed that the high risk of CVD in DM patients is due to oxidative modifications of low-density lipoproteins (LDL), and this model has also been extended to HDL. Arising from these hypotheses, numerous clinical trials have assessed the ability of antioxidants in the primary and secondary prevention of CVD in DM [3,32]. The most common antioxidant used in these trials was vitamin E, which has been classified as a potent antioxidant with an ability to scavenge free radicals and singlet oxygen [33,34]. Vitamin E was of particular interest for three main reasons. Firstly, it can be reduced by other antioxidants, thus preventing the accumulation of vitamin E radicals. Secondly, it has a well-understood mechanism as to how it prevents the oxidation of LDL [35]. Lastly, there was strong evidence in preclinical models and observational studies in humans that vitamin E treatment could reduce CVD [3,36].

Observational studies attempting to associate vitamin E levels with CVD found that individuals with high serum levels of vitamin E had a lower incidence of CVD [37]. However, randomized placebo-controlled studies assessing the ability of vitamin E to reduce the incidence of CVD in individuals with DM have failed to show a benefit of vitamin E in reducing CVD incidence in DM individuals [38]. Several meta-analyses of these studies have shown that vitamin E may be associated with a 5–10% increased rate of death in individuals with DM. 

One suggestion that was proposed to explain the failure of vitamin E in the prevention of CVD is that vitamin E would only benefit those patients under extreme antioxidant stress, and the previous trials had not differentiated between groups of patients [3,37]. The SPACE trial showed that patients with high levels of oxidative stress due to end-stage renal disease greatly benefited from vitamin E antioxidant therapy [3].

As previously shown, DM individuals with the Hp2-2 genotype have increased levels of oxidative markers, suggesting that Hp 2-2 individuals are under a higher degree of oxidative stress. This led to the hypothesis that vitamin E antioxidant therapy could benefit DM patients with the Hp2-2 genotype [3,39]. This hypothesis was directly assessed in three trials. In the HOPE study, 9541 men and women were randomized to receive treatment with vitamin E and/or Ramipril or a placebo for 4.5 years, and no clinical benefit from vitamin E was observed. However, in a retrospective analysis of those HOPE participants, segregated by Hp type, whose serum was stored at baseline, vitamin E treatment significantly reduced the risk of MI and CVD death by more than 40% and 50%, respectively [40]. Serum levels had been taken at the onset of the studies to eliminate a survivorship bias. 

The ICARE study treated 1434 DM Hp2-2 individuals with vitamin E or placebo, attempting to validate the results of the HOPE study. After 18 months, patients in the vitamin E treatment group demonstrated significantly lower incidences of stroke, MI, and CVD-related death rates, compared to patients in the placebo group [41].

The Women’s Health Study was designed to assess whether vitamin E prevented CVD over 10 years in non-diabetic and diabetic women and it found no benefit of vitamin E. However, in a retrospective analysis of diabetic WHS participants whose sera had been stored, vitamin E administration was associated with a non-significant 14% reduction in CVD events. 

## 5. Strict Glycemic Control in Patients with the Hp2-2 Genotype Provides CV Benefit

Hyperglycemia has been proposed to play a major role in the development of diabetic complications. Interventions designed to reduce hyperglycemia in the DCCT and United Kingdom Prospective Diabetes Study (UKPDS) studies demonstrated that treating hyperglycemia could reduce the incidence of the microvascular complications of DM (retinopathy and nephropathy) [42,43]. However, these studies did not demonstrate a significant benefit to CVD. Multiple, more recent studies (Action to Control Cardiovascular Risk in Diabetes (ACCORD), Action in Diabetes and Vascular Disease: Preterax and Diamicron MR Controlled Evaluation (ADVANCE), and Veterans Affairs Diabetes Trial (VADT)) attempting to reduce CVD in DM via aggressive glycemic control have failed in their aim. In the ACCORD study, there were 10,251 participants with a mean age of 62. They had a median baseline of HbA_1c_ of 8.1%. In the study with intensive glycemic control therapy, the investigators attempted to lower patients’ HbA_1c_ levels to below 6%. They compared their intervention with standard glycemic control aiming to reduce HbA_1c_ levels to between 7% and 7.9%. The intensive glycemic control therapy reached a median HbA_1c_ level of 6.4%, while the standard glucose therapy group reached a median HbA_1c_ level of 7.5%. Although the intensive glycemic control therapy nearly reached the targeted HbA_1c_ levels, in February of 2008, the glycemic control study of ACCORD was halted due to an increase in mortality rate compared to that in the standard-therapy group [18]. The investigators were unable to identify the cause of this increase in mortality rate [44]. The outcomes of the ACCORD study were nonfatal MI, nonfatal stroke, and CVD death, as shown in Table 2, and no CHD benefit from aggressive glycemic control could be demonstrated [18]. The ADVANCE study randomized 11,140 participants from Europe, New Zealand, Canada, and Asia. They compared intensive glycemic control with standard glycemic control. In the ADVANCE study, compared to the ACCORD study, the participants were slightly older (all above the age of 55, with a mean age of 66 compared to a mean age of 62 in the ACCORD study). The ADVANCE study had a lower baseline median HbA_1c_ level of 7.2%. The intensive glycemic control group and the standard glycemic control group showed HbA_1c_ levels of 6.3% and 7%, respectively. 

The primary outcome of the ADVANCE comprised both microvascular events, including nephropathy and retinopathy, and major adverse cardiovascular events, including MI, stroke, and cardiovascular death. Aggressive glycemic control demonstrated no significant clinical benefit for the combined endpoint of microvascular and macrovascular disease or for macrovascular disease alone [18]. In the Veterans Affairs Diabetes Trial (VADT), 1791 type 2 DM veterans had a mean age of 60 and a median baseline HbA1c level of 9.4%. They were randomized to intensive or conventional glycemic control. The median HbA1c levels were reduced by the intensive glycemic control therapy and by the standard glycemic control therapy to 6.9% and 8.5%, respectively [18]. The outcomes of the VADT trial included MI, stroke, cardiovascular death, revascularization, heart failure, and amputation for ischemia. The intensive glycemic control therapy group showed an insignificantly higher rate of cardiovascular death compared to the standard glycemic control group [18]. 

We have proposed that benefits from strict glycemic control may only exist in those individuals, such as those with the Hp 2-2 genotype, in whom hyperglycemia confers a greater risk of CVD [6]. In two cohorts with of HbA1c levels in broad ranges—the ICARE and the NHS—it was shown that Hp2-2 genotype individuals with HbA1c concentrations >6.5% had a tenfold increased incidence of coronary heart disease, compared to non-Hp2-2 individuals with HbA1c concentrations <6.5% [45]. Similarly, in the HPFS which had 39% of participants with the Hp2-2 genotype, Hp2-2 individuals showed greater risks of CHD in the presence of hyperglycemia [46].

Since the Hp 2-2 genotype confers an increased risk of CVD only in those Hp 2-2 individuals in whom HbA1c is elevated (greater than 6.5%), we proposed that studies in which strict glycemic control failed to show benefit would have shown benefit if the intervention was limited to only those patients with the Hp 2-2 genotype [6]. In the ACCORD study, assessing the role of strict glycemic control in nearly 10,000 DM individuals, glycemic control was found to provide significant benefit only in Hp 2-2 DM individuals and to be associated with harm in non-Hp 2-2 individuals. The investigators compared results of Hp1 carriers with those of Hp2-2 carriers, both groups receiving intensive or standard therapy, as shown in Table 2. Regarding coronary heart disease in Hp1 carriers, 12.2% of these patients presented CHD events when under intensive therapy, compared with 13.3% of patients who were administered the standard therapy.

Patients with the Hp2-2 genotype showed 10.3% and 13.8% rates of CHD when receiving intensive therapy and standard therapy, respectively. Regarding CVD, 9.6% of Hp1 carriers receiving intensive therapy and 10.7% of those administered standard therapy had CVD events. On the other hand, Hp2-2 patients in the intensive therapy group and standard therapy group had CVD events rates of 8.7% and 11.5%, respectively. The total mortality rate of Hp1 carriers was 7.4% for patients in the intensive therapy group and 5.5% for those in the standard therapy group. Patients with the Hp2-2 genotype presented a 5.6% mortality rate if in the intensive therapy group and a 5.4% mortality rate if in the standard therapy group. It should be noted that the rates of CHD and CVD were both significantly reduced in Hp2-2 participants treated with intensive glucose reduction therapy. No such benefit of intensive glycemic control on CVD was seen in Hp1 carriers. Regarding the total rates of mortality, on the other hand, it can be seen here that there was an increase in total mortality in Hp1 patients treated with intensive glucose reduction therapy [6].

## 6. Limitations and Future Directions

Collectively, the precision medicine-based approach based on vitamin E administration to Hp 2-2 DM individuals derived from the retrospective analysis of the HOPE and WHS studies, coupled with that of the prospective ICARE study, has been applied to a relatively small number of patients. Before this algorithm can be recommended for clinical use, a much larger placebo-based randomized clinical trial should be performed [3]. While the public health significance of and healthcare savings derived from the validation of the Hp genotype–vitamin E-based approach for precision medicine would be profound, a significant barrier to carrying out such a larger study in the current medical research environment is the lack of financial incentive for big pharmaceutical companies for research on vitamin E [3].

Regarding the analysis of the Hp genotype to determine if glycemic control will be beneficial, it is clear that the ACCORD findings will need to be extended to other clinical trial cohorts that have assessed the clinical benefit of tight glycemic control, such as those of VADT and ADVANCE [6]. Furthermore, as the ACCORD Hp analysis was performed only in Caucasians, it will be important to determine if this finding is consistent across different ethnic groups. One complication of performing such analysis in African Americans is the presence of a fourth Hp phenotype, the Hp 2-1 modified genotype, which is found in around 10% of all African Americans [47]. Prospective trials investigating the Hp-based glycemic control algorithm will also be necessary before this can be accepted clinically as a treatment guideline [6]. However, if validated, this glycemic control algorithm would be of great benefit, helping to control health care resources and manpower. Nevertheless, as mentioned above for vitamin E, the incentive for big pharmaceutical companies to conduct such a study is limited.

In addition to vitamin E and glycemic control, there may exist other simple inexpensive interventions that may reduce the risk of CVD in Hp 2-2 DM individuals. The Look AHEAD study assessed the ability of the modification of risk factors including smoking, high weight, and nutrition to improve CVD outcomes in DM patients and failed to show any benefit [48]. It would be very interesting to investigate if benefit would be seen in Hp 2-2 DM individuals in this trial. By conducting Hp genotyping and screening for Hp2-2, further studies could obtain significant clinical results in reducing the risk of CVD. 

## Figures and Tables

**Table 1:** Studies assessing incident cardiovascular disease in relation to the haptoglobin genotype. OR is the ratio of disease for Hp 2-2 carriers compared to Hp 1 carriers [13]. HOPE; Heart Outcome Prevention Evaluation, SHS, Strong Heart Study, ICARE, Israel Cardiovascular Vitamin E, EDC, Epidemiology of Diabetes Complications, WHS, Women’s Health Study, Diabetes Heart Study (DHS), DCCT/EDIC, Diabetes Control and Complications Trial/Epidemiology of Diabetes Interventions and Complications, NHS, Nurses’ Health Study, HPFS, Health Professionals Follow-Up Study.

Study	Sample Size		Outcomes	OR (95% CI)
	Hp2-2	Hp1 Carrier		
HOPE Study	91	187	Myocardial infarction (MI), stroke, cardiovascular (CV) death, and all-cause death	2.183 (1.223–3.896)
SHS	66	173	MI and CV events	2.507 (1.325–4.743)
MUNICH post-PCA	382	553	Death, target vessel revascularization (TVR), MI, repeat percutaneous transcatheter coronary angioplasty (PTCA), and restenosis	1.364 (1.022–1.821)
ICARE study	1434	1533	CV death, all-cause death, TVR, MI, stoke, and heart failure	1.714 (1.087–2.704)
EDC Study	214	239	MI, revascularization, stenosis >50%, or death from coronary artery disease (CAD)	1.542 (1.029–2.312)
WHS	277	444	Nonfatal MI, nonfatal stroke, cardiovascular death, PTCA, or coronary artery bypass grafting (CABG)	1.209 (0.776–1.884)
DHS	535	673	CV mortality and all-cause mortality	1.599 (1.060–2.413)
DCCT/EDIC	516	787	CAD and MI	1.244 (0.882–1.755)
NHS	26	44	Nonfatal MI and fatal CHD	4.000 (0.811–19.728)
Bruneck Study	387	419	Incident fatal and nonfatal MI and stoke	0.960 (0.654–1.410)
HPFS	38	50	Nonfatal MI or fatal CHD	1.253 (0.475–3.305)
Overall	3966	5102		1.44 (1.23–1.69)

**Table 2:** Assessment of the effect of aggressive versus conventional glycemic control therapy on cardiovascular outcomes and total mortality in the ACCORD study [6].

Strict Glycemic Control																
	CHD				CVD				Fatal CVD				Total Mortality			
Genotype	Hp1 Carrier		Hp2-2		Hp1 Carrier		Hp2-2		Hp1 Carrier		Hp2-2		Hp1 Carrier		Hp2-2	
Treatment	Intensive Therapy	Standard Therapy	Intensive Therapy	Standard Therapy	Intensive Therapy	Standard Therapy	Intensive Therapy	Standard Therapy	Intensive Therapy	Standard Therapy	Intensive Therapy	Standard Therapy	Intensive Therapy	Standard Therapy	Intensive Therapy	Standard Therapy
Percent of Participants with Events	12.2% (*n* = 224)	13.3% (*n* = 244)	10.3% (*n* = 110)	13.8% (*n* = 147)	9.6% (*n* = 175)	10.7% (*n* = 197)	8.7% (*n* = 92)	11.5% (*n* = 123)	3.2% (*n* = 58)	2.2% (*n* = 40)	2.5% (*n* = 27)	2.3% (*n* = 25)	7.4% (*n* = 135)	5.5% (*n* = 101)	5.6% (*n* = 59)	5.4% (*n* = 58)
adjusted Hazards Ratio (aHR) (95% CI)	0.95 (0.79–1.13)		0.91 (0.75–1.13)		0.91 (0.75–1.13)		0.71 (0.54–0.93)		1.50 (1.00–2.25)		1.02 (0.59–1.77)		1.40 (1.08–1.81)		0.98 (0.68–1.41)	
*p* Value	*p* = 0.550		*p* = 0.392		*p* = 0.392		*p* = 0.013		*p* = 0.049		*p* = 0.931		*p* = 0.011		*p* = 0.908	

## Abbreviations

Hp	Haptoglobin
Hb	Hemoglobin
DM	Diabetes Mellitus
CVD	Cardiovascular Disease
ACCORD	Trial Action to Control Cardiovascular Risk in Diabetes Trial
VADT	Trial Veterans Affairs Diabetes Trial
ADVANCE	Trial Action in Diabetes and Vascular Disease: Preterax and Diamicron MR Controlled Evaluation Trial
